# Kidney-Tonifying, Phlegm-Resolving, and Blood Stasis–Removing Therapy for Multiple Myeloma: Protocol for a Randomized Controlled Trial on Epigenetic and Immune Modulation

**DOI:** 10.2196/86322

**Published:** 2026-03-05

**Authors:** Xiaoqi Sun, Yongming Zhou, Yujue Wang, Youya Dai, Wenwei Zhu, Hailin Chen

**Affiliations:** 1 Department of Hematology, Yueyang Hospital of Integrated Traditional Chinese and Western Medicine, Shanghai University of Traditional Chinese Medicine, 110 Ganhe Road, Hongkou District, Shanghai, 200437, China, 86 13651784266

**Keywords:** myeloma, herbal medicine, randomized controlled trial, epigenetic regulation, tumor immune microenvironment

## Abstract

**Background:**

Multiple myeloma (MM) is characterized by kidney deficiency, phlegm, and blood stasis as core findings, specifically in Traditional Chinese Medicine (TCM), and the kidney-tonifying, phlegm-resolving, and blood stasis–removing (KPR) method is a fundamental therapeutic approach for MM in TCM. Western medicine primarily focuses on targeted immunotherapy or chemotherapy for MM treatment, whereas TCM characterizes MM through distinct pathological patterns that directly correspond to immune microenvironment dysregulation. Emerging evidence implicates the PHD finger protein 19 (PHF19)/enhancer of zeste homolog 2 (EZH2)/trimethylated histone H3 at lysine 27 (H3K27me3) epigenetic axis in immune microenvironment dysregulation and MM progression. Notably, TCM “blood stasis” correlates with hypoxia-induced immune gene silencing in MM bone marrow, and KPR (a clinically validated TCM decoction with 16 herbs) acts on this axis via its active components that regulate EZH2 and epigenetic function, merging TCM syndrome differentiation with modern epigenetics. We have designed a randomized controlled trial (RCT) to investigate the mechanism of action and safety of the KPR method in MM.

**Objective:**

This RCT aims to assess whether a KPR herbal formula combined with standard bortezomib-based therapy improves the immune microenvironment via the PHF19-EZH2-H3K27me3 epigenetic axis to restore immune function in MM, providing a mechanistic basis for integrating TCM into evidence-based oncology care in relapsed or refractory patients.

**Methods:**

This is a single-center, prospective RCT involving patients with MM. It has been designed to test the hypothesis that the KPR formula epigenetically regulates the PHF19-EZH2-H3K27me3 axis to improve the immune microenvironment. Patients are randomly assigned in a 1:1:1 ratio to 3 groups (blank control group, Western medicine control group, and integrated TCM and Western medicine treatment group). All patients undergo 12 weeks of treatment and a 6-month follow-up. The primary outcome is the CD3^+^ T-cell ratio in bone marrow/peripheral blood, which is detected by flow cytometry. The secondary outcomes include quantified TCM syndrome scores, Western medicine efficacy evaluation criteria, complete blood count, bone marrow morphology, blood and urine immunoglobulin levels, quantitative M protein levels, free light chain levels, β2-microglobulin levels, and whole-body imaging findings. Statistical analysis involves linear mixed models for longitudinal data and Bonferroni correction to verify KPR’s immunomodulatory effects via the targeted epigenetic axis.

**Results:**

This study was funded in November 2023. Recruitment was initiated in March 2025 and is expected to be completed in February 2026. As of October 2025, 41 patients have been enrolled. Data collection is projected to end in October 2026. Data analysis has not yet been initiated, and the results are expected to be published in 2027.

**Conclusions:**

This unique mechanistic RCT evaluating a TCM formula targeting the PHF19-EZH2-H3K27me3 axis in patients with MM will establish a biomarker-driven framework for integrating TCM with immunotherapy, offering novel strategies for treatment-refractory patients.

## Introduction

### Background

Multiple myeloma (MM) is a hematological malignancy driven by clonal plasma cell proliferation, which damages multiple organ systems. Its main manifestations include bone destruction, renal impairment, anemia, hypercalcemia, and infection [1]. Since 2010, immunotherapy has initiated a revolutionary era of treatment for MM [2,3]. Currently, many novel targets for the treatment of specific MM subtypes have been clinically identified, significantly improving progression-free survival and overall survival [4-9]. However, MM remains incurable, and drug resistance inevitably develops [10-12].

PHD finger protein 19 (PHF19) expression strongly correlates with MM progression and shows superior predictive value over the high-risk gene *MMSET*. Knockout of PHF19 causes myeloma cell lines to enter a state of reduced proliferation. As a key component of polycomb repressive complex 2 (PRC2) [13], PHF19 is expressed across all MM subgroups and is particularly overexpressed in high-risk patients with MM. Existing studies have shown that increased histone methylation, especially H3K27 trimethylation, is closely associated with disease aggressiveness [14]. PHF19 regulates histone methylation to modulate chromatin activity [15] and facilitates B cell differentiation into plasma cells in germinal centers [16]. The mechanisms of PHF19 in MM biology require further investigation. Another critical methyltransferase in MM is enhancer of zeste homolog 2 (EZH2), a PRC2 subunit. EZH2 primarily methylates H3K27, silencing target genes and contributing to MM onset, progression, and poor prognosis [17]. While proteasome inhibitor therapy can counteract its adverse effects, EZH2’s negative prognostic impact appears treatment-independent [18]. Beyond driving tumorigenesis via histone methylation, EZH2 also disrupts immune homeostasis to shape the tumor microenvironment (TME), promoting progression [19]; however, its specific effects on the TME in MM require further exploration [20]. Functionally, PHF19 is closely linked to EZH2, regulating PRC2/EZH2 recruitment and activity to modulate genes critical for cell growth and differentiation. PHF19 thereby influences cell cycle and genetic stability genes, correlating strongly with high-risk MM behavior [21].

In summary, PHF19 and EZH2 are closely related. As a key epigenetic methyltransferase, EZH2 participates in the pathogenesis within the TME. These findings suggest that the triad of PHF19, EZH2, and the immune microenvironment interacts and contributes to the pathological mechanisms of multidrug resistance in MM. However, related research on this topic has not been thoroughly investigated.

In ancient Chinese medicine, MM was not specifically named but was categorized based on symptoms such as “bone bi syndrome” (骨痹), “low back pain” (腰痛), “consumptive disorder” (虚劳), and “bone erosion” (骨蚀). At the 2008 Special Symposium on TCM Disease Names for Hematological Diseases, a consensus was reached to designate “marrow tumor” (骨髓瘤) as the Traditional Chinese Medicine (TCM) disease name for MM [22]. However, physicians have held varying views on its etiology and pathogenesis [23,24]. The primary pathogenic factors are generally attributed to “toxin” (毒), “deficiency” (虚), “phlegm” (痰), and “blood stasis” (瘀). Toxin invasion weakens the body’s vital energy (正气), leading to the generation of phlegm and stasis; this interplay of underlying deficiency and superficial pathogenic factors creates a vicious cycle of mutual reinforcement. Yueyang Hospital of Integrated Traditional Chinese and Western Medicine (affiliated to the Shanghai University of Traditional Chinese Medicine) has explored treatment approaches combining Western medicine with the “method of tonifying the kidney, resolving phlegm, and removing blood stasis.” The basic formula consists of the following components: *Taxillus chinensis* (24 g), *Eucommia ulmoides* (24 g), *Epimedium* herb (24 g), stir-fried *Atractylodes macrocephala* rhizome (12 g), *Scrophularia ningpoensis* root (12 g), processed *Pinellia ternata* rhizome (18 g), *Prunella vulgaris* spike (15 g), white mustard seed (9 g), *Fritillaria thunbergii* bulb (18 g), oyster shell (30 g), *Hedyotis diffusa* herb (30 g), *Scutellaria barbata* herb (30 g), *Duchesnea indica* herb (15 g), *Eupolyphaga sinensis* insect (9 g), processed *Bombyx batryticatus* larva (9 g), and processed *Glycyrrhiza uralensis* root (6 g). Preliminary qualitative mass spectrometric analysis by our research group identified active components, including naringenin, icariin, and formononetin, which demonstrate therapeutic efficacy against MM [25-27]. Rooted in clinical experience, this approach addresses the core pathogenesis and variable manifestations of MM, showing good patient acceptance.

However, the mechanism by which the kidney-tonifying, phlegm-resolving, and blood stasis–removing (KPR) method combined with a basic Western medicine regimen treats MM has not been thoroughly investigated. Therefore, we propose the following hypothesis: The KPR method may act on PHF19 to regulate EZH2/trimethylated histone H3 at lysine 27 (H3K27me3) epigenetically, ultimately improving the immune microenvironment to inhibit immune escape and exert therapeutic effects, and thereby enhancing the clinical efficacy of MM.

### Aims and Research Questions

We propose a multilevel mechanistic framework bridging TCM syndrome differentiation with PHF19-EZH2-H3K27me3–mediated immune dysregulation in MM.

First, we think kidney deficiency (肾虚) indicates PHF19 transcriptional activation. In TCM, the “kidney” governs the “marrow” and governs “essence” (精), analogous to hematopoietic stem cell niche integrity [28]. We think that kidney deficiency–induced stress signals may transcriptionally activate PHF19. Herbs, such as *Scutellaria barbata* and *Scutellaria baicalensis*, may stabilize HIF-1α degradation through prolyl hydroxylase domain (PHD) inhibition, thereby suppressing PHF19 overexpression in MM cells [29-31].

Second, we think phlegm indicates EZH2 catalytic activity. “Phlegm” in MM corresponds to pathological glycoprotein-rich microenvironment and extracellular matrix remodeling. Phlegm-induced transforming growth factor-β (TGF-β) signaling enhances EZH2 phosphorylation at Ser21, increasing its methyltransferase activity [32,33]. Phlegm-resolving herbs, such as rhizome of lagehead atractylodes, may inhibit TGF-β phosphorylation, blocking EZH2 recruitment to the PRC2 complex and thus reducing H3K27me3 deposition on immune gene loci [34,35].

Third, blood stasis may indicate H3K27me3-mediated immune gene silencing. Stasis reflects hypoxia, acidosis, and inflammatory cytokine entrapment in MM bone marrow. This microenvironment induces DNA hypermethylation at CpG islands of T-cell exhaustion genes, which synergizes with H3K27me3 and irreversibly silences effector function [36,37]. Stasis-eliminating herbs, such as *Hedyotis diffusa* and ground beetle *Eupolyphaga sinensis*, may inhibit EZH2 interaction, preventing heterochromatin propagation.

Finally, we think the KPR formula induces immune reprogramming through polypharmacological effects, such as direct PHF19 siRNA-like activity via herb-derived microRNAs [38-40] and gut microbiome modulation by herbs to increase *Lactobacillus* [41].

This hypothesis integrates TCM “holistic network regulation” with molecular epigenetics, providing a falsifiable mechanistic scaffold for our randomized controlled trial (RCT).

## Methods

### Study Location and Recruitment Procedure

The study is conducted at the Department of Hematology, Yueyang Hospital of Integrated Traditional Chinese and Western Medicine, Shanghai University of Traditional Chinese Medicine, a tertiary hospital with integrated TCM-Western medicine expertise, ensuring targeted recruitment of patients with MM seeking care here.

**Figure 1.**
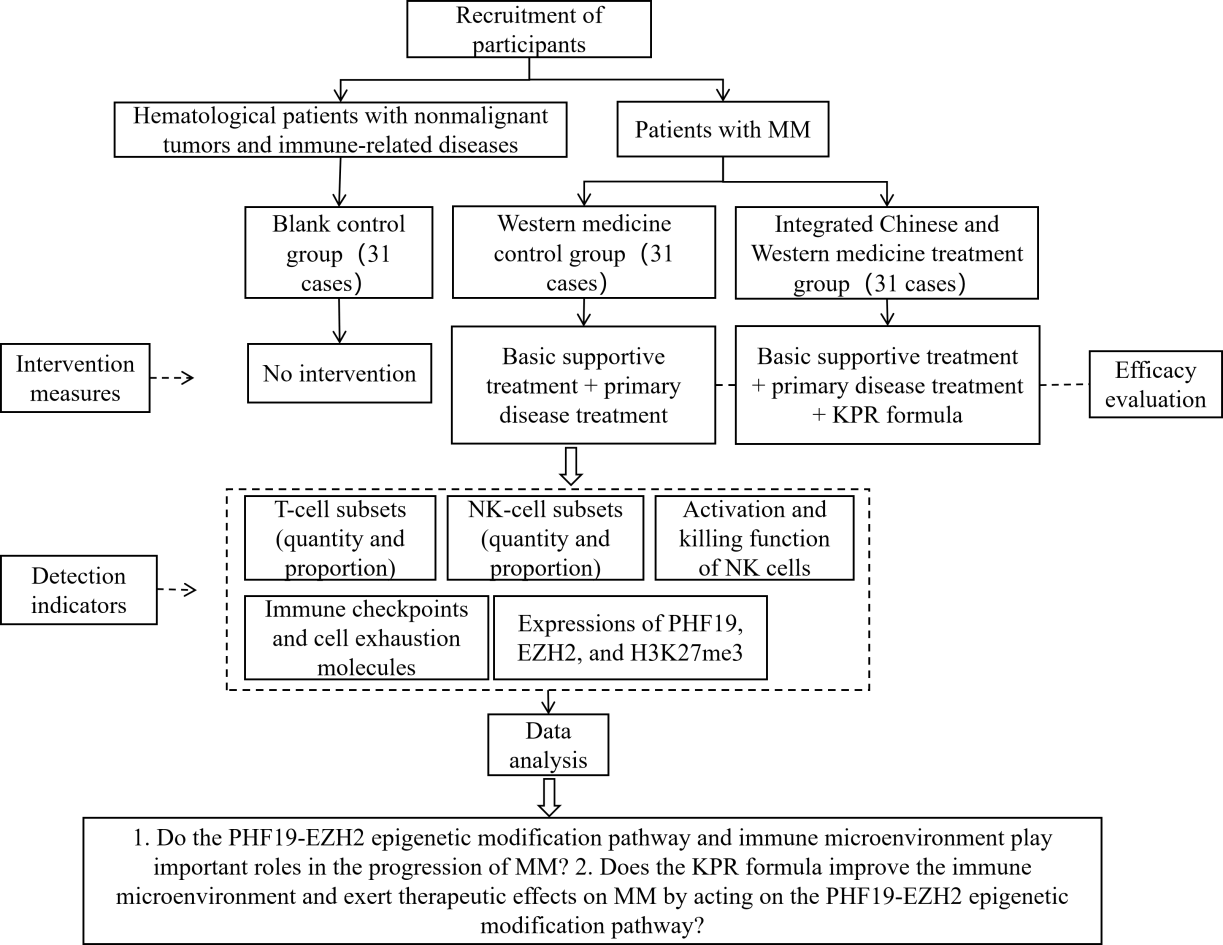
Study flowchart. EZH2: enhancer of zeste homolog 2; H3K27me3: trimethylated histone H3 at lysine 27; KPR: kidney-tonifying, phlegm-resolving, and blood stasis–removing; MM: multiple myeloma; NK: natural killer; PHF19: PHD finger protein 19.

Recruitment follows a systematic, multichannel procedure. Public advertisements are posted on electronic display screens in hematology clinic waiting areas and MM/hematological disease patient education WeChat groups. Eligible interested individuals contact the dedicated research team via the advertised phone number or in-person inquiries during clinic visits, undergo preliminary screening with a standardized checklist (eg, age and MM diagnosis), and complete a comprehensive formal evaluation (Western medical MM diagnosis confirmation, TCM syndrome differentiation, and exclusion criteria review), and those meeting all criteria are enrolled after receiving detailed study information and signing a written informed consent form. The SPIRIT (Standard Protocol Items: Recommendations for Interventional Trials) checklist is provided in Checklist 1. The study flowchart is shown in Figure 1.

### Source of Cases and Study Population

A total of 62 patients with MM meeting the diagnostic criteria have been enrolled from the outpatient and inpatient departments of hematology at Yueyang Hospital of Integrated Traditional Chinese and Western Medicine. Additionally, 31 patients with hematological diseases (excluding malignancies and immune-related disorders) have been included in a blank control group.

### Diagnostic Criteria

#### Western Medicine Diagnostic Criteria

Diagnosis was based on the following criteria: a bone marrow monoclonal plasma cell proportion ≥10% and/or histopathological confirmation of plasmacytoma, with at least one of the SLiM-CRAB features (Multimedia Appendix 1).

#### TCM Diagnostic Criteria

The condition falls under the category of “bone bi syndrome” (骨痹) in TCM. The TCM syndrome differentiation was formulated with reference to the Diagnostic and Therapeutic Criteria for Internal Medicine Diseases and Syndromes in TCM issued by the National Administration of TCM [42], combined with the common clinical manifestations of patients with MM. The TCM syndrome diagnostic criteria for kidney deficiency, phlegm, and blood stasis syndrome (Multimedia Appendix 2) have been applied, requiring the presence of all major symptoms and at least 2 secondary symptoms, and confirmation via tongue and pulse examinations. All 3 TCM practitioners have undergone standardized training. Interrater reliability for syndrome diagnosis is κ=0.78 (substantial agreement) [22-24]. TCM diagnosis retains inherent subjectivity despite standardized criteria and substantial interrater reliability (κ=0.78). We have mitigated this by correlating syndrome improvements with objective molecular markers (PHF19 and H3K27me3).

### Inclusion, Exclusion, and Discontinuation Criteria

The inclusion, exclusion, and discontinuation criteria are provided in Textbox 1.

Textbox 1. **Inclusion, exclusion, and discontinuation criteria.**

**Inclusion criteria**

- Diagnosis of multiple myeloma (MM) according to Western medicine diagnostic criteria
- Diagnosis of kidney deficiency, phlegm, and blood stasis syndrome (TCM syndrome) based on TCM diagnostic criteria
- Age ≥18 years or ≤80 years, with no gender restriction
- Willingness to receive the treatment regimen and sign an informed consent form

**Exclusion criteria**

- Allergic constitution or hypersensitivity to MM therapeutic agents
- Severe cardiovascular diseases, including myocardial infarction or heart failure of grade 3 or higher
- Severe abnormalities in liver or renal function (alanine aminotransferase >5 times the upper limit of normal; glomerular filtration rate <15 mL/min)
- Clinically significant systemic diseases, such as osteoporosis, bone metastatic carcinoma, or lumbar tuberculosis
- Presence of ≥2 complications (eg, bone disease, infection, hemorrhage, and anemia)
- Alcoholism, drug abuse, mental disorders, or intellectual disability
- Pregnancy or lactation

**Discontinuation, termination, and attrition criteria**

- Inability to tolerate the trial drug
- Development of serious adverse events or adverse reactions related to the treatment regimen
- Withdrawal by the patient (eg, due to poor efficacy or intolerable adverse reactions)
- Poor compliance (eg, failure to take medication as prescribed, addition of other medications that can affect efficacy assessment, or failure to attend follow-up visits)
- Loss to follow-up

### Randomization and Blinding

Randomization numbers are assigned based on the order of patient visits, and participants are grouped using the random number table method.

The KPR herbal decoction is prepared using herbs supplied by Shanghai Hongqiao TCM Co, Ltd, and uniformly decocted by the pharmacy of Yueyang Hospital of Integrated Traditional Chinese and Western Medicine, Shanghai University of Traditional Chinese Medicine, under standardized parameters (200 g of herbs boiled in 1600 mL of water for 45 minutes at 100 °C; concentrated to 200 mL per pouch). All batches are required to meet safety standards for heavy metals and pesticides. Patients receive sequential batches per enrollment order, with batch numbers documented for potential sensitivity analysis. A placebo, primarily composed of maltodextrin and lactose, is color-matched with caramel to mimic the appearance and weight of the active decoction.

This is a single-blind trial. Participants remain blinded to allocation; the placebo matches the KPR decoction in appearance, taste, and packaging (maltodextrin base with added natural coloring and bitter flavoring). Outcome assessors (laboratory staff and statisticians) are blinded, while TCM practitioners who dispense decoctions and adjust prescriptions based on syndrome differentiation are not blinded for clinical feasibility. Blinding integrity will be assessed using the Bang Index at weeks 4 and 12.

### Grouping and Interventions

#### Overview

A total of 62 patients with MM are randomly divided into the following 2 groups: Western medicine control group and integrated TCM-Western medicine treatment group, with 31 patients in each group. The general clinical data of the 2 groups are comparable. Additionally, 31 patients with nonmalignant and nonimmune hematological diseases are included in a blank control group (Multimedia Appendix 3).

Given that MM is a life-threatening malignancy, a pure placebo arm would be ethically indefensible according to the Helsinki Declaration §33 [43]. Our design includes the following study arms:

- Blank control (arm 1): Patients with nonmalignant hematological diseases providing baseline immune reference values, which are essential for the mechanistic interpretation of PHF19-EZH2 dynamics in cancer versus normal states.
- Western medicine control (arm 2): Patients with MM receiving standard-of-care bortezomib-based therapy, which serves as an active comparator, enabling the isolation of the additive effect of KPR therapy on immune reprogramming.
- Integrated TCM-Western medicine (arm 3): Patients with MM receiving the test intervention.

This design directly addresses our hypothesis: Does KPR therapy augment immune recovery beyond what is achieved by standard therapy alone? A placebo-KPR group would answer a different question (placebo effect magnitude) but would not inform clinical integration.

#### Blank Control Group

Patients receive treatment for underlying diseases as needed, with no additional interventions administered.

#### Western Medicine Control Group

Patients receive risk-stratified supportive care according to Padua thrombotic risk assessment scores (Multimedia Appendix 4) combined with standard antimyeloma therapy (Multimedia Appendix 5).

#### Integrated TCM-Western Medicine Treatment Group

Patients receive basic supportive care, treatment for the primary disease, and KPR therapy. The modification of prescriptions based on syndrome differentiation is shown in Multimedia Appendix 6.

### Data Collection and Management

Data are collected from daily symptom records and examination forms completed by participants, as well as from case report forms (CRFs) and TCM syndrome scores documented by medical staff and laboratory results obtained from Yueyang Hospital of Integrated Traditional Chinese and Western Medicine.

CRFs are double-entered and cross-checked by trained clinical research coordinators/data managers. Paper CRFs are transcribed within 7 days. Monitors conduct on-site source data verification. The system is preset with logical rules: key indicators are 100% verified, and nonkey indicators are spot-checked at a rate of ≥20%. Data discrepancies are automatically flagged, generating a query list sent to investigators for resolution within 5 days, and unresolved issues are escalated to monitors. After prelocking, the database is reviewed by the Clinical Trial Data Monitoring Committee. Final locking requires confirmation by the sponsor, investigators, and statisticians, with data use exclusively for statistical analysis. Electronic data will be encrypted, backed up daily, and retained for ≥5 years. Paper documents will be stored in locked cabinets, archived within 3 months after the trial, and retained for ≥10 years.

Investigators have completed Good Clinical Practice (GCP) and protocol training before enrollment. Routine monitoring includes on-site visits every 4 weeks to verify CRF quality and source data. Centralized monitoring via the electronic data capture system enables real-time detection of anomalies. Annual third-party audits assess GCP compliance.

Before unblinding, a comprehensive review of the data is conducted to evaluate the appropriateness of defining the intention-to-treat population, modified intention-to-treat population, per-protocol population, and safety analysis population. This assessment requires joint approval from the data manager, study promoter, and core researchers. Unblinding is completed after database locking, formally initiating the statistical analysis and results reporting phase.

### Assessment: Primary and Secondary Endpoints

#### Primary Endpoints

Flow cytometry is used to detect the CD3^+^ T-cell ratio (primary outcome) in bone marrow and peripheral blood, and assess other T-cell subsets, including CD4^+^ T cells, CD8^+^ T cells, and CD4^+^CD25^+^FOXP3^+^ regulatory T cells (Tregs). Moreover, the immune checkpoint molecules PD-1, LAG-3, and TIM-3 are evaluated. Furthermore, natural killer (NK) cell subsets, including CD3⁻CD56^+^ NK cells, CD56^high^CD16⁻ NK cells, and CD56^dim^CD16^+^ NK cells, are assessed, and the presence of the activation molecule NKG2D on NK cells and the expression levels of its ligands (MICA and MICB) on CD34^+^ cells are determined. In addition, the expression levels of NK cytotoxic activity markers CD107a, interferon-γ, granzyme B, and perforin are evaluated.

#### Secondary Endpoints

For quantifying the TCM syndrome score, syndromes (main symptoms, secondary symptoms, and tongue and pulse manifestations) are observed and recorded once before treatment and at 12 weeks after treatment. The efficacy is graded as follows: (1) complete recovery (disappearance or near-disappearance of clinical symptoms and signs, with a reduction in the syndrome score of ≥95%), (2) marked improvement (significant improvement in clinical symptoms and signs, with a reduction in the syndrome score of 70%‐95%), (3) effective (improvement in clinical symptoms and signs, with a reduction in the syndrome score of 30%‐70%), and (4) ineffective (no significant improvement in clinical symptoms and signs, with a reduction in the syndrome score of <30%). The nimodipine method is used for calculation, and the formula is as follows: ([pretreatment score − posttreatment score] / pretreatment score) × 100%.

For the assessment of Western medicine, efficacy is evaluated according to the 2024 Revised Chinese Guidelines for the Diagnosis and Treatment of MM at 12 weeks after treatment and is categorized as follows: (1) stringent complete response, (2) complete response, (3) very good partial response, (4) partial response, (5) minimal response, (6) stable disease, (7) progressive disease, and (8) clinical relapse.

The main symptoms include generalized bone pain, fever, fatigue, dysuria, nausea, vomiting, numbness, and tingling in the hands and feet. The secondary symptoms include soreness and weakness in the lower back and knees, shortness of breath, dizziness, tinnitus, palpitations, chest tightness, hemorrhage, edema, and loss of appetite. The tongue and pulse manifestations include tortuous sublingual veins, a pale and dull tongue, a white and slippery coating, and a deep, weak pulse. Observations and records are made before and after treatment.

Blood tests include complete blood count and C-reactive protein. Bone marrow assessments include bone marrow cytology, bone marrow biopsy for pathological analysis, chromosome analysis, flow cytometry, and abnormal gene testing. Blood and urine are assessed for immunoglobulins, quantitative M protein, free light chains, and β2-microglobulin. Furthermore, whole-body radiography, computed tomography, magnetic resonance imaging, and positron emission tomography-computed tomography are performed. Observations are made before treatment and at 12 weeks after treatment.

### Safety Evaluation

Safety evaluations, including urinalysis/stool routine, liver/renal function tests, and electrocardiography, are conducted at baseline and at 12 weeks after treatment.

### Adverse Event Record

Adverse event time, type (eg, adverse reaction and device malfunction), and severity (mild, moderate, or severe); basic subject information (name abbreviation and ID); management measures (eg, drug discontinuation and symptomatic treatment); and outcomes (recovery, persistence, or death) of adverse events or serious adverse events (SAEs) are recorded.

Adverse events are documented within 24 hours using standardized forms, and these are reviewed and signed by the principal investigator with clinical research coordinator assistance and Ethics Committee oversight. SAEs are reported to the Ethics Committee and regulatory authorities within 24 hours. Non-SAEs are reported quarterly. Both paper (signed) and electronic (encrypted) records will be archived for ≥5 years. Potential adverse reactions include Western medicine–related bone marrow suppression (anemia, leukopenia, thrombocytopenia, hemorrhage, and infection/sepsis), gastrointestinal toxicity (nausea, vomiting, abdominal pain, diarrhea, and mucosal ulcers), hepatotoxicity (elevated transaminases, fatty liver, and jaundice), dermatological reactions (dermatitis, drug rash, and phlebitis), and disease progression/relapse, as well as KPR method–related gastrointestinal side effects (mild diarrhea, nausea, and anorexia from cold/bitter herbs like *Oldenlandia diffusa* and *Scutellaria barbata*) and allergic reactions (rashes, papules, and erythema) to herbs such as *Eupolyphaga sinensis* and *Bombyx batryticatus*.

### Sample Size Calculation

This study is an RCT, and the CD3^+^ T-cell ratio of participants is the observed outcome indicator. Based on a comprehensive review of the literature, the CD3^+^ T-cell ratio in the Western medicine control group is determined to be 50.15 (SD 4.27). It is estimated that the CD3^+^ T-cell ratio in the integrated TCM-Western medicine treatment group could increase by 3.86 points [44,45]. With a 2-tailed α of .05 and a power of 90%, the sample size is calculated using the following formula:

$n=\frac{2(Z_{\alpha }+Z_{\beta }{)}^{2}*\sigma^{2}}{\delta }$

where Z_α_/2 is the critical value for a 2-tailed test at α=.05 (1.96), Z_β_ is the critical value for a power of 90% (1.28), σ is the SD (4.27), and δ is the difference in means (3.86).

Using the CD3+ T-cell ratio as the observed outcome indicator, with a 2-tailed α of .05 and a power of 90%, the required sample size per group is 26 cases. Considering 1:1:1 randomization, 26 cases are needed per group. Accounting for a 15% loss to follow-up and refusal, a minimum of 31 cases per group (total ≥93 cases) are to be enrolled.

While the MM immune microenvironment is indeed complex, we have selected the CD3^+^ T-cell ratio as the sole primary outcome for sample size calculation based on the following integrated rationale.

CD3^+^ T cells have a central integrator function. They serve as the master regulator of adaptive antitumor immunity. Their abundance in bone marrow directly correlates with clinical response to immunotherapy and overall survival in patients with MM (ρ=0.71; *P*<.01) [1]. As a composite marker, CD3^+^ T-cell recovery integrates successful reprogramming of multiple upstream pathways (Treg suppression, checkpoint blockade, and NK-T cell crosstalk), making it a more robust single indicator than isolated subset markers.

CD3^+^ T cells have a direct mechanistic linkage to the PHF19-EZH2 axis. PHF19 overexpression specifically drives T-cell exhaustion through H3K27me3-mediated silencing of T-cell effector genes [40]. Our preclinical data show that PHF19 knockdown increases CD3^+^ T-cell infiltration by 4.2-fold, mirroring the hypothesized 3.86-point clinical effect. Thus, CD3^+^ T cells represent the most proximal and relevant indicator of our targeted epigenetic intervention.

According to CONSORT (Consolidated Standards of Reporting Trials) guidelines, a single primary outcome avoids multiplicity inflation (type I error) and ensures adequate power for the core hypothesis. The secondary endpoints characterize the full immune landscape without requiring separate powering, and if used, they would require an over 3-fold larger sample size, compromising the feasibility of this early phase mechanistic trial.

Food and Drug Administration guidance for immuno-oncology mechanistic trials supports the use of a single validated immune parameter for powering early phase studies, with composite endpoints reserved for confirmatory trials [46,47].

### Statistical Analysis

To address the study hypothesis that the KPR formula epigenetically regulates the PHF19-EZH2-H3K27me3 axis to improve the immune microenvironment, descriptive statistics, including number, percentage, mean, and SD, will be used to describe variables as appropriate. Normally distributed continuous data will be summarized as mean (SD), while nonnormally distributed continuous data will be presented as median and IQR. Categorical data will be expressed as frequency and percentage.

For the primary outcome (CD3^+^ T-cell ratio) and key mechanistic indicators (PHF19, EZH2, and H3K27me3 expression levels), one-way ANOVA will be used for 3-group comparisons (blank control, Western medicine control, and integrated TCM-Western medicine treatment) if the data are normally distributed and homogeneous in variance; otherwise, the Kruskal-Wallis H test will be applied. Post hoc pairwise comparisons will use Bonferroni correction to control for type I error. For other continuous outcomes with normal distribution, the independent samples *t* test will be used for 2-group comparisons, and for outcomes with nonnormal distribution, the Mann-Whitney *U* test will be used. For categorical outcomes, the chi-square test or Fisher exact test will be used when expected cell counts are less than 5. Intragroup comparisons before and after treatment will utilize the paired samples *t* test for normally distributed data and the Wilcoxon signed-rank test for nonnormally distributed data.

Longitudinal data analysis across multiple time points will be performed using linear mixed models or generalized estimating equations to account for correlations between repeated measurements from the same participant. These models will include “treatment group” (fixed effect), “time point” (fixed effect), and “group×time interaction” (fixed effect) to test whether the KPR formula induces sustained improvements in the primary outcome and mechanistic indicators beyond standard therapy, efficiently handling unequal time intervals and missing data under the missing-at-random assumption.

When multiple hypothesis tests are performed simultaneously, Bonferroni correction will be applied to control for type I error risk by adjusting the significance level. The pattern and mechanism of missing data will first be assessed, and if missing data are minimal (less than 5%), complete-case analysis may be used, while larger proportions will be handled using multiple imputation methods to create several complete datasets for analysis with pooled results. Moreover, sensitivity analyses will be conducted to evaluate the impact on conclusions.

To further validate the mechanistic link in the hypothesis, Pearson or Spearman correlation analysis will be used to explore the association between PHF19/EZH2/H3K27me3 expression levels and the CD3^+^ T-cell ratio in the integrated TCM-Western medicine group, verifying whether the KPR formula exerts immunomodulatory effects through the targeted epigenetic axis.

All analyses will be performed on an intention-to-treat basis. A *P* value of <.05 is considered to indicate statistical significance, unless otherwise adjusted for multiple comparisons.

### Ethical Considerations

#### Ethics Committee Approval

This study has been approved by the Ethics Committee of Yueyang Hospital of Integrated Traditional Chinese and Western Medicine, Shanghai University of Traditional Chinese Medicine (ethics approval number: 2025-006; approval date: February 10, 2025). The study protocol is conducted in accordance with the Declaration of Helsinki and relevant international ethical guidelines for human subjects research.

#### Informed Consent

The study protocol and potential risks/benefits are fully explained to all eligible participants. Written informed consent is obtained from each participant before enrollment and specimen collection. Participants have the right to withdraw from the study at any time without penalty, and their decision will not affect their subsequent medical care.

#### Privacy and Confidentiality

All participant information is deidentified using coded ID numbers, with no personal identifiers (eg, name and contact information) linked to study records. Documents containing personal identifiers (eg, informed consent forms and locator forms) are stored separately from deidentified study data in locked cabinets with restricted access. Electronic data are encrypted, password-protected, and backed up daily. Laboratory specimens, reports, and administrative forms are labeled only with coded IDs. All study-related records (paper and electronic) will be retained for ≥5 years (electronic) or ≥10 years (paper), as required by regulatory standards, and will not be disclosed to third parties without written permission from participants.

#### Participant Compensation

No financial or material compensation is provided to participants for their involvement in this study.

#### Patient and Public Involvement

Patients and/or the public are not involved in the design, conduct, reporting, or dissemination plans of this research.

## Results

The study was funded in November 2023, with recruitment of eligible patients initiated in March 2025. Eligibility assessment targeted patients with MM meeting Western medical diagnostic criteria and TCM kidney deficiency, phlegm, and blood stasis syndrome criteria. As of October 2025, 41 patients with MM have been enrolled, with follow-ups scheduled according to the study protocol. Recruitment is projected to be completed by February 2026, with a target enrollment of ≥93 patients. Data collection is expected to conclude in October 2026, and the study results are anticipated to be published in 2027.

## Discussion

### Anticipated Main Findings

We hypothesize that KPR therapy will significantly enhance the CD3^+^ T-cell ratio by 3.86 points versus bortezomib-based standard therapy, selected as the primary endpoint for integrating Treg suppression, checkpoint modulation, and NK crosstalk. Beyond this, we anticipate improvements in (1) immunological parameters (reduced Treg proportions, decreased checkpoint molecule expression, and enhanced NK cytotoxicity), (2) clinical outcomes (better TCM syndrome scores and higher stringent complete response/complete response rates), and (3) safety (no excess adverse events). Confirmation would provide the first clinical evidence that TCM-mediated epigenetic modulation of the PHF19-EZH2-H3K27me3 axis reprograms the MM immune microenvironment.

### Principal Findings in Relation to Prior Research

Our results, if positive, would suggest that KPR therapy can counteract EZH2-driven immune suppression. This builds directly on preclinical observations indicating that PHF19 overexpression elevates immune checkpoint molecules and Treg cell frequencies [13,14,48]. These findings form the mechanistic basis for our hypothesis that a multiherb formula can epigenetically modulate the MM microenvironment, potentially shifting it from an immune-evasive state to a more immune-permissive state. The anticipated improvement in CD3^+^ T-cell function is particularly significant, given that T-cell exhaustion constitutes a major barrier to immunotherapy efficacy in MM [1]. Our 3-arm design (lacking an independent KPR placebo) is justified by the pragmatic framework requiring standard therapy and a mechanistic focus on augmentation. The Western medicine control serves as a benchmark to attribute improvements to KPR’s epigenetic modulation vs spontaneous recovery, aligning with FDA integrative oncology guidance [44].

### Comparison With Prior Work

Unlike prior TCM MM studies focused on palliation or synergistic cytotoxicity [22-24], our study is the first to test a biomarker-driven mechanism (PHF19-EZH2-H3K27me3 axis). If validated, KPR may offer a multitarget, lower-toxicity alternative to single-agent epigenetic inhibitors (eg, EZH2 inhibitors) [18], and our blank control group (nonmalignant hematological diseases) provides a critical immunological baseline rarely established in MM trials.

### Strengths and Limitations

The key strengths of our study include its prospective randomized controlled design, single-blind allocation, and comprehensive immune monitoring using flow cytometry. The novel hypothesis connecting TCM principles with epigenetic-immune crosstalk represents a potential paradigm shift in integrative oncology research. However, several limitations must be acknowledged. For the first time, this study proposes the concept that Chinese herbal medicine with the therapeutic principle of “tonifying the kidney, resolving phlegm, and eliminating stasis” improves the clinical efficacy of MM by acting on PHF19 expression to mediate epigenetic regulation via the EZH2/H3K27me3 pathway, ultimately enhancing the immune microenvironment. This provides new therapeutic ideas and directions for MM treatment. There is currently a lack of international consensus on the use of TCM for the treatment of MM. Powering the analysis solely on the CD3^+^ T-cell ratio may not capture the full immune complexity, and this is mitigated by comprehensive secondary profiling and planned mediation analysis, though future trials should consider composite immune scores for powering. Additionally, a single-blind design is necessary because practitioners cannot be blinded due to TCM syndrome differentiation requirements, which may introduce performance bias. To mitigate this, we will use blinded outcome assessors who are independent of the treatment team and unaware of group allocation. Furthermore, a standardized base formula will be used to minimize variability in TCM prescriptions.

### Future Directions

This protocol lays the groundwork for several important translational next steps. Positive findings would warrant validation in larger, multicenter cohorts with extended follow-up to assess the impacts on progression-free and overall survival. Mechanistic studies using techniques, such as mass spectrometry, could help identify the active components within the KPR formula that directly modulate the PHF19-EZH2 interaction. Combination studies with novel immunotherapies (eg, bispecific antibodies and chimeric antigen receptor [CAR] T cells) could explore whether KPR therapy helps overcome resistance mechanisms by restoring T-cell fitness. Finally, developing a "PHF19 expression signature" could enable better patient stratification for personalized TCM-integrated treatment approaches.

### Dissemination Plan

Consistent with open-science principles, we plan to disseminate our findings through multiple channels as follows: (1) publication in peer-reviewed journals within the fields of oncology and integrative medicine; (2) presentation at major international conferences (eg, American Society of Hematology Annual Meeting and International Myeloma Society Workshop); (3) deposition of deidentified immune monitoring datasets in public repositories (eg, Zenodo) to facilitate secondary analyses; and (4) collaboration with TCM practitioner networks to develop evidence-based clinical practice guidelines, should the efficacy of the approach be demonstrated.

### Conclusions

This protocol describes the first rigorous investigation of whether a TCM formula can epigenetically reprogram the MM immune microenvironment via the PHF19-EZH2-H3K27me3 axis. By bridging mechanistic biomarker research with clinical outcomes, this study may establish a scientifically grounded framework for integrating TCM into evidence-based MM management, offering new hope for patients who develop resistance to conventional therapies.

## Supplementary material

10.2196/86322 Multimedia Appendix 1 Descriptions of CRAB and SLiM.

10.2196/86322 Multimedia Appendix 2 Syndrome differentiation of kidney deficiency and phlegm stasis pattern in Traditional Chinese Medicine.

10.2196/86322 Multimedia Appendix 3 Group differences.

10.2196/86322 Multimedia Appendix 4 Padua score.

10.2196/86322 Multimedia Appendix 5 Basic disease treatment.

10.2196/86322 Multimedia Appendix 6 Modification of prescriptions based on syndrome differentiation.

10.2196/86322 Checklist 1 SPIRIT checklist.

## Abbreviations

CRF: case report form
EZH2: enhancer of zeste homolog 2
GCP: Good Clinical Practice
H3K27me3: trimethylated histone H3 at lysine 27
KPR: kidney-tonifying, phlegm-resolving, and blood stasis–removing
MM: multiple myeloma
NK: natural killer
PHF19: PHD finger protein 19
PRC2: polycomb repressive complex 2
RCT: randomized controlled trial
SAE: serious adverse event
TCM: Traditional Chinese Medicine
TGF-β: transforming growth factor-β
TME: tumor microenvironment
Treg: regulatory T cell
