# Supplementary material for: Kidney-Tonifying, Phlegm-Resolving, and Blood Stasis–Removing Therapy for Multiple Myeloma: Protocol for a Randomized Controlled Trial on Epigenetic and Immune Modulation
Source: JMIR Res Protoc. 2026 Mar 5;15:e86322. doi: 10.2196/86322 (PMC12978978; doi:10.2196/86322)
Supplement: Multimedia Appendix 1 [file resprot-v15-e86322-s001.docx]

**Multimedia Appendix 1.** Descriptions of CRAB and SLiM.

| Name | Definition |
| --- | --- |
| [C] | Corrected serum calcium >2.75 mmol/L [Corrected serum calcium (mmol/L) = Total serum calcium (mmol/L) – 0.025 × Serum albumin concentration (g/L) + 1.0 (mmol/L); or Corrected serum calcium (mg/dL) = Total serum calcium (mg/dL) – Serum albumin concentration (g/L) + 4.0 (mg/dL)] |
| [R] | Renal impairment (creatinine clearance <40 mL/min or serum creatinine >177 μmol/L) |
| [A] | Anemia (hemoglobin below the lower normal limit by 20 g/L or <100 g/L) |
| [B] | Osteolytic destruction (imaging studies [X-ray, CT, or PET-CT] show ≥1 osteolytic lesion) |
| [S] | Bone marrow monoclonal plasma cell proportion ≥60%! |
| [Li] | Ratio of involved/uninvolved serum free light chains ≥100 (Involved light chain value ≥100 mg/L) |
| [M] | MRI detects >1 focal bone lesion >5 mm |
