# Supplementary material for: Kidney-Tonifying, Phlegm-Resolving, and Blood Stasis–Removing Therapy for Multiple Myeloma: Protocol for a Randomized Controlled Trial on Epigenetic and Immune Modulation
Source: JMIR Res Protoc. 2026 Mar 5;15:e86322. doi: 10.2196/86322 (PMC12978978; doi:10.2196/86322)
Supplement: Multimedia Appendix 2 [file resprot-v15-e86322-s002.docx]

**Multimedia Appendix 2.** Syndrome differentiation of kidney deficiency and phlegm stasis pattern in Traditional Chinese Medicine.​

| Main Symptoms | Secondary Symptoms | Tongue Manifestations | Pulse Manifestations |
| --- | --- | --- | --- |
| Lumbosacral or hypochondriac pain | Weakness and soreness of the waist and knees | Tortuosity of sublingual collateral vessels | Deep and weak pulse |
| Fever | Shortness of breath | Pale and dull tongue | Wiry or slippery pulse |
| Fatigue | Hemorrhage | White and slippery coating | / |
| / | Dizziness and tinnitus | / | / |
