# Supplementary material for: Kidney-Tonifying, Phlegm-Resolving, and Blood Stasis–Removing Therapy for Multiple Myeloma: Protocol for a Randomized Controlled Trial on Epigenetic and Immune Modulation
Source: JMIR Res Protoc. 2026 Mar 5;15:e86322. doi: 10.2196/86322 (PMC12978978; doi:10.2196/86322)
Supplement: Multimedia Appendix 3 [file resprot-v15-e86322-s003.docx]

**Multimedia Appendix 3.** Group differences.

| Group | Number of Participants | Population | Intervention Measures | Specific Methods | Treatment Duration |
| --- | --- | --- | --- | --- | --- |
| Blank Control Group | 31 cases | Patients with non-malignant tumors and immune-related hematological diseases | No intervention. | / | / |
| Western Medicine Group | 31 cases | Patients with MM | Basic supportive care + primary disease treatment. | Bortezomib/Lenalidomide/Dexamethasone (Vrd) regimen: 21 days as 1 treatment cycle. Specific medications: - Bortezomib: 1.3 mg/m² subcutaneous injection on days 1, 4, 8, 11; - Dexamethasone: 20 mg intravenous drip on days 1, 2, 4, 5, 8, 9, 11, 12; - Lenalidomide: 25 mg oral administration on days 1–14. | 4 treatment cycles (12 weeks in total), followed by a 24-week follow-up. |
| Integrated Chinese-Western Medicine Group | 31 cases | Patients with MM | Basic supportive care + primary disease treatment + kidney- tonifying, phlegm- resolving and stasis-eliminating therapy. | Same as the western medicine control group for basic supportive care and primary disease treatment. The kidney-tonifying, phlegm-resolving and stasis-eliminating therapy is modified based on a basic herbal formula. All herbal ingredients are sourced from Yueyang Hospital pharmacy, compliant with the 2000 Chinese Pharmacopoeia, centrally decocted by the hospital pharmacy, concentrated twice, yielding 200 ml of decoction. Administered twice daily (morning and evening), 100 ml each time. | 4 treatment cycles (12 weeks in total), followed by a 24-week follow-up. |
