# Supplementary material for: Kidney-Tonifying, Phlegm-Resolving, and Blood Stasis–Removing Therapy for Multiple Myeloma: Protocol for a Randomized Controlled Trial on Epigenetic and Immune Modulation
Source: JMIR Res Protoc. 2026 Mar 5;15:e86322. doi: 10.2196/86322 (PMC12978978; doi:10.2196/86322)
Supplement: Multimedia Appendix 4 [file resprot-v15-e86322-s004.docx]

**Multimedia Appendix 4.** Padua score.

| Risk Factors | Score |
| --- | --- |
| Active malignant tumor, with local or distant metastasis and/or chemotherapy/radiotherapy within 6 months | 3 |
| Previous venous thromboembolism (VTE) | 3 |
| Immobility (patient bedridden for ≥3 days due to physical reasons or medical orders) | 3 |
| Thrombophilia (antithrombin deficiency, protein C/S deficiency, factor V Leiden mutation, prothrombin G20210A mutation, antiphospholipid antibody syndrome) | 3 |
| Recent (≤1 month) trauma or surgery | 2 |
| Age ≥70 years | 1 |
| Heart failure and/or respiratory failure | 1 |
| Acute myocardial infarction and/or ischemic stroke | 1 |
| Acute infection and/or rheumatic disease | 1 |
| Obesity (body mass index ≥35 kg/m²) | 1 |
| Ongoing hormone therapy | 1 |
