# Supplementary material for: Kidney-Tonifying, Phlegm-Resolving, and Blood Stasis–Removing Therapy for Multiple Myeloma: Protocol for a Randomized Controlled Trial on Epigenetic and Immune Modulation
Source: JMIR Res Protoc. 2026 Mar 5;15:e86322. doi: 10.2196/86322 (PMC12978978; doi:10.2196/86322)
Supplement: Multimedia Appendix 5 [file resprot-v15-e86322-s005.docx]

**Multimedia Appendix 5.** Basic disease treatment.

| Name | Classification | Medication | Dosage and Administration |
| --- | --- | --- | --- |
| Bone Disease | Normal Renal Function | Zoledronic Acid | 15mg, Intravenous Drip, Once Monthly |
|  | Renal Insufficiency | Denosumab | 120mg, Subcutaneous Injection |
| ​​Infection | / | Sensitive Antibiotics | As Needed |
| ​​Bleeding | / | Hemostatic Drugs, VitC, Platelets <20×10⁹/L with obvious bleeding: Transfusion of Apheresis Platelet Suspension | As Needed |
| ​​Severe Anemia | / | Hemoglobin <60g/L: Transfusion of Red Blood Cell Suspension | As Needed |
| ​​Granulocytopenia | Neutrophils <0.5×10⁹/L | Granulocyte Colony-Stimulating Factor (G-CSF) or Granulocyte-Macrophage Colony-Stimulating Factor (GM-CSF) | 5–10μg/(kg·d), Subcutaneous Injection |
| ​​Renal Protection via Hydration and Alkalinization | / | Sodium Bicarbonate Tablets | 0.5g per dose, Twice Daily, Orally |
| ​​Anticoagulation | Low-Risk | Aspirin (Baia Aspirin) | 100mg per dose, Once Nightly, Orally |
|  | High-Risk | Low-Molecular-Weight Heparin | 1 vial per dose, Once Daily, Subcutaneous |

According to the Chinese Expert Consensus on Mechanical Prophylaxis of Venous Thromboembolism (2020 Edition), non-surgical patients should use the Padua Score for venous thromboembolism risk assessment: low-risk (0–3 points), high-risk (>4 points). Initiate stratified prophylactic anticoagulation or antiplatelet therapy when the score is ≥0 (refer to Table S1).
