# Supplementary material for: Kidney-Tonifying, Phlegm-Resolving, and Blood Stasis–Removing Therapy for Multiple Myeloma: Protocol for a Randomized Controlled Trial on Epigenetic and Immune Modulation
Source: JMIR Res Protoc. 2026 Mar 5;15:e86322. doi: 10.2196/86322 (PMC12978978; doi:10.2196/86322)
Supplement: Multimedia Appendix 6 [file resprot-v15-e86322-s006.docx]

**Multimedia Appendix 6.** Modification of prescriptions based on syndrome differentiation.

| Syndrome Pattern/Symptoms | Modifications |
| --- | --- |
| Damp-Heat Syndrome | Cangzhu, Huangbai |
| Pale complexion, dizziness and tinnitus, palpitations, shortness of breath, spontaneous sweating on exertion, low voice, fatigue and weakness | Astragalus, Salvia miltiorrhiza, Rehmannia glutinosa |
| Dry stools | Rhubarb |
| Severe pain, like needle pricking | Sanleng, Ezhu |
| Dry mouth | Trichosanthes root, Rehmannia |
| Severe whole-body pain, unbearable, unable to sleep at night | Corydalis Rhizoma, Boswellia Carterii, Pollen Typhae, Bletilla striata |
